# *In situ* phenotypic heterogeneity among single cells of the filamentous bacterium *Candidatus* Microthrix parvicella

**DOI:** 10.1038/ismej.2015.181

**Published:** 2015-10-27

**Authors:** Abdul R Sheik, Emilie EL Muller, Jean-Nicolas Audinot, Laura A Lebrun, Patrick Grysan, Cedric Guignard, Paul Wilmes

**Affiliations:** 1 Luxembourg Centre for Systems Biomedicine, University of Luxembourg, Esch-sur-Alzette, Luxembourg; 2 Materials Research and Technology Department, Luxembourg Institute of Science and Technology, Belvaux, Luxembourg; 3 Department of Environmental Research and Innovation, Luxembourg Institute of Science and Technology, Belvaux, Luxembourg

## Abstract

Microorganisms in biological wastewater treatment plants require adaptive strategies to deal with rapidly fluctuating environmental conditions. At the population level, the filamentous bacterium *Candidatus* Microthrix parvicella (*Ca*. M. parvicella) has been found to fine-tune its gene expression for optimized substrate assimilation. Here we investigated *in situ* substrate assimilation by single cells of *Ca*. M. parvicella using nano-scale secondary-ion mass spectrometry (nanoSIMS). NanoSIMS imaging highlighted phenotypic heterogeneity among *Ca*. M. parvicella cells of the same filament, whereby ^13^C-oleic acid and ^13^C-glycerol-3-phosphate assimilation occurred in ≈21–55% of cells, despite non-assimilating cells being intact and alive. In response to alternating aerobic–anoxic regimes, ^13^C-oleic acid assimilation occurred among subpopulations of *Ca.* M. parvicella cells (≈3–28% of cells). Furthermore, *Ca*. M. parvicella cells exhibited two temperature optima for ^13^C-oleic acid assimilation and associated growth rates. These results suggest that phenotypic heterogeneity among *Ca*. M. parvicella cells allows the population to adapt rapidly to fluctuating environmental conditions facilitating its widespread occurrence in biological wastewater treatment plants.

Activated sludge-based biological wastewater treatment plants (BWWTPs) rely on the substrate assimilation capabilities of microorganisms to drive metabolic transformations culminating in wastewater remediation (Daims *et al.*, 2006). Frequent changes in the influent substrate composition and variations in environmental factors as well as alternating aerobic and anoxic phases result in BWWTPs representing highly fluctuating environments. Therefore, microbial populations in BWWTPs require adaptive strategies to deal with these continuous perturbations.

Laboratory-based studies have suggested that phenotypic heterogeneity among individual cells of isogenic populations confers adaptive advantages in fluctuating environments (De Jong *et al.*, 2011; Levy *et al.*, 2012). Phenotypic heterogeneity may reflect a bet-hedging strategy whereby multiple phenotypes of isogenic populations constitute a series of bets in response to rapidly changing environmental conditions (Levy *et al.*, 2012). In particular population-level variations in the expression of genes involved in carbon assimilation allows populations to hedge their bets (De Jong *et al.*, 2012). Single-cell approaches allow the study of within-population phenotypic heterogeneity (Grimbergen *et al.*, 2015). Nano-scale secondary-ion mass spectrometry (nanoSIMS), which allows visualization and quantification of differences in substrate assimilation among individual microbial cells, is particularly well suited for this task (Zimmermann *et al.*, 2015).

*Candidatus* Microthrix parvicella (*Ca.* M. parvicella) is a ubiquitous lipid-accumulating filamentous bacterium that can dominate municipal BWWTPs resulting in operational difficulties, such as sludge bulking and foaming (Rossetti *et al.*, 2005). Based on laboratory, *in situ* and genomic investigations, *Ca.* M. parvicella appears to be metabolically versatile and can assimilate diverse carbon substrates while being adaptable to a wide range of environmental conditions, for example, oxygen concentrations and temperatures (Andreasen and Nielsen, 1998; Tandoi *et al.*, 1998; Nielsen *et al.*, 2002; Muller *et al.*, 2012; McIlroy *et al.*, 2013). A previous *in situ* microautoradiographic study has highlighted differences in substrate assimilation among *Ca.* M. parvicella filaments (Kindaichi *et al.*, 2013). At the population-level, recent community-wide integrated omic analyses indicate that *Ca.* M. parvicella exhibits varying levels of expression for genes involved in substrate assimilation (primarily long-chain fatty acids; Muller *et al.*, 2014a) but exhibits overall low levels of genetic variation (McIlroy *et al.*, 2013; Muller *et al.*, 2014a). Based on these observations, we hypothesized that phenotypic heterogeneity among individual *Ca*. M. parvicella cells might be a mechanism for the population to adapt to the rapidly changing environmental conditions encountered in BWWTPs.

Here we investigated substrate assimilation by *Ca*. M. parvicella cells using ^13^C-oleic acid, ^13^C-triolein, ^13^C-glycerol and ^13^C-glycerol-3-phosphate. Four independent time-series incubation experiments were performed each in duplicate (Figure 1a, details in Supplementary Methods). Single-cell substrate assimilation of *Ca.* M. parvicella was quantified using a combination of fluorescence *in situ* hybridization and nanoSIMS (Figures 1b and c) as well as bulk stable isotopic analyses using liquid chromatography coupled to tandem mass spectrometry (Supplementary Table S1). Furthermore, we verified the integrity and cellular morphology of *Ca.* M. parvicella cells and filaments using atomic force microscopy (Figures 1b and c).

First, we investigated potential fine-scale differences in the fatty acid assimilation of ^13^C-triolein and ^13^C-oleic acid under aerobic and anoxic conditions. The ^13^C-oleic acid assimilation rates by *Ca*. M. parvicella cells were most pronounced under anoxic conditions after 1 h of incubation (Figure 1d), underlining the preference of microaerophilic conditions by *Ca*. M. parvicella (Rossetti *et al.*, 2005). Thereafter, the highest rates of assimilation were attained under both aerobic and anoxic conditions after 5 h followed by a significant reduction (analysis of variance, *P*<0.0001) by 8 h of the experiment (Figure 1d). Importantly, ^13^C-oleic acid remained detectable in the supernatant fraction of the experimental samples (Supplementary Table S1) and, thus, the observed trend was not due to exhaustion of the substrate over time. In contrast to ^13^C-oleic acid, ^13^C-triolein assimilation by *Ca*. M. parvicella cells was minimal (Supplementary Figure S1).

The seasonal dominance of *Microthrix* populations during wintertime has been partially attributed to the higher bioavailability of lipid substrates when wastewater temperatures are lower (Rossetti *et al.*, 2005; Muller *et al.*, 2014a; Roume *et al.*, 2015). By taking into account that ^13^C-oleic acid assimilation by *Ca*. M. parvicella was highest after 5 h with equal assimilation rates under aerobic or anoxic conditions, we performed temperature-dependent incubation experiments under aerobic conditions over a wide range of temperatures (4–35 °C), and we then compared *Ca*. M. parvicella ^13^C-oleic acid assimilation rates at the 5 h time point (Figure 1e). ^13^C-oleic acid assimilation was apparent at 4 °C but markedly decreased with increasing temperatures (4–20 °C). Between 25 and 30 °C, ^13^C-oleic acid assimilation increased significantly (analysis of variance, *P*<0.0001) but decreased again at 35 °C. The observed two temperature optima may be attributed to differences in the bioavailability of ^13^C-oleic acid (higher levels of bioavailability are expected at the lower temperatures, for example, at 4 °C; Rossetti *et al.*, 2005) and altered activity of the acyl-CoA ligases for ^13^C-oleic acid assimilation (higher assimilation rates might be expected at the higher temperatures, for example, 25 °C). These wide ranges of temperature-dependent ^13^C-oleic acid assimilation characterized by two temperature optima emphasize *Ca.* M. parvicella's generalist lifestyle strategy (Muller *et al.*, 2014a), defined as an ability to tolerate a wide range of environmental conditions.

*Ca.* M. parvicella encodes glycerol and glycerol-3-phosphate transporters (McIlroy *et al.*, 2013) and can simultaneously assimilate oleic acid and glycerol (Kindaichi *et al.*, 2013). Recent genome-scale metabolic reconstructions suggest that glycerol conversion into glycerol-3-phosphate may occur prior to its assimilation (McIlroy *et al.*, 2013; Roume, 2013). To investigate these phenotypic traits, we carried out experiments using ^13^C-glycerol or ^13^C-glycerol-3-phosphate in combination with or without unlabeled oleic acid. Interestingly, *Ca.* M. parvicella cells assimilated ^13^C-glycerol-3-phosphate only as a single substrate measurable after 8 and 24 h of the experiment under both aerobic and anoxic conditions (Figure 1f). Although the absence of ^13^C-glycerol assimilation is consistent with a previous study (Tomei *et al.*, 1999), the lack of simultaneous assimilation with oleic acid is at odds with the observations of another *in situ* study (Kindaichi *et al.*, 2013), which may suggest intraspecific phenotypic differences according to geographic location. Nonetheless, the rapid assimilation of ^13^C-oleic acid compared with ^13^C-glycerol-3-phosphate underlines previous suggestions that *Ca.* M. parvicella engages in optimal foraging behavior (Muller *et al.*, 2014a), which posits that, in an environment with diverse substrates, successful taxa will have a preference for the most energy-dense substrates (Frens, 2010).

Intriguingly, nanoSIMS imaging revealed extensive phenotypic heterogeneity in substrate assimilation between individual *Ca.* M. parvicella cells of the same filament (Figure 2). For instance, ≈35–55% and ≈5–35% of *Ca.* *M*. parvicella cells assimilated ^13^C-oleic acid and ^13^C-glycerol-3-phosphate, respectively, whereas the remainder of cells (45–95%) did not exhibit any ^13^C-substrate assimilation (Supplementary Table S2). Furthermore, phenotypic heterogeneity in the ^13^C-oleic acid assimilation appeared to be temperature-dependent whereby relatively low phenotypic heterogeneity was observed at 4 and 30 °C, respectively (Supplementary Table S2). To date, nanoSIMS imaging of filamentous bacteria from other environments has revealed variations in substrate assimilation among cells of the same population (Popa *et al.*, 2007; Vasquez-Cardenas *et al.*, 2015). However, the complete absence of ^13^C-substrate assimilation in a substantial fraction of cells belonging to the same filament is unique to the results presented in this study. Importantly, intense fluorescence *in situ* hybridization signals, atomic force microscopic cell integrity results acquired prior to nanoSIMS analyses and Live-Dead staining (Boulos *et al.*, 1999; Roume *et al.*, 2013) did not reveal differences in terms of viability between assimilating and non-assimilating cells, suggesting that the observed intercellular phenotypic heterogeneity is an intrapopulation feature of *Ca.* M. parvicella (Figures 1a–c, Supplementary Figure S2).

We further estimated *Ca.* M. parvicella growth rates based on cells that exhibited substrate assimilation (Foster *et al.*, 2011) as much of newly assimilated ^13^C-oleic acid appeared to be utilized for cell growth rather than for triglyceride accumulation as ^13^C-glyceryl trioleate (Supplementary Table S2). In response to different substrates and temperature conditions tested in this study, the estimated *Ca.* M. parvicella growth rates ranged from 0.12 to 0.78 day^−1^, which are in agreement with those estimated using the total extended filament length approach (Tandoi *et al.*, 1998; Rossetti *et al.*, 2002).

Given the prevalence of *Ca.* M. parvicella phenotypic heterogeneity, we investigated *Ca.* M. parvicella ^13^C-oleic acid assimilation in response to alternating aerobic–anoxic phases, a regularly encountered fluctuation in BWWTPs in which *Ca.* M. parvicella can become prominent. In response to alternating anoxic phases, ≈28% of aerobically preconditioned *Ca.* M. parvicella cells exhibited a wider range of ^13^C-oleic acid assimilation rates compared with ≈3% of anoxically preconditioned *Ca.* M. parvicella cells which experienced alternating aerobic conditions (Figure 1g). Compared to their non-alternated controls, less ^13^C-oleic acid assimilation was observed among *Ca.* M. parvicella cells subjected to alternating conditions (Supplementary Figure S3). This was reflected in the presence of subpopulations of assimilating *Ca.* M. parvicella cells, which in turn suggests that an increase in phenotypic heterogeneity (Supplementary Table S2) results from fluctuating environmental conditions and reflects a possible adaptation strategy. Given the low levels of population-level genetic variation in *Ca.* M. parvicella (McIlroy *et al.*, 2013; Muller *et al.*, 2014a) as well as the expected clonality among cells of the same filament, genetic variation is unlikely to be the source for observed phenotypic heterogeneity among *Ca.* M. parvicella cells. However, the observed phenotypic heterogeneity among subpopulations of *Ca.* M. parvicella cells suggests that this population follows a bet-hedging strategy.

The adaptive function of phenotypic heterogeneity has been well described in laboratory studies, yet its significance in natural and engineered environments is poorly understood. Here we provide direct evidence for phenotypic heterogeneity among cells of *Ca*. M. parvicella that is independent of varied ^13^C-oleic acid assimilation rates in response to different temperature and alternating aerobic–anoxic regimes (Figures 1d, e and g, and Supplementary Figure S3). Given that *Ca.* M. parvicella intermittently blooms resulting in operational difficulties (Rossetti *et al.*, 2005) or that it may represent a means of recovering chemical energy in the form of lipids from wastewater (Muller *et al.*, 2014b), strategies for controlling its growth in BWWTPs are highly desirable. Our results highlight the importance of accounting for phenotypic heterogeneity in devising such schemes in the future.

## Figures and Tables

**Figure 1:**
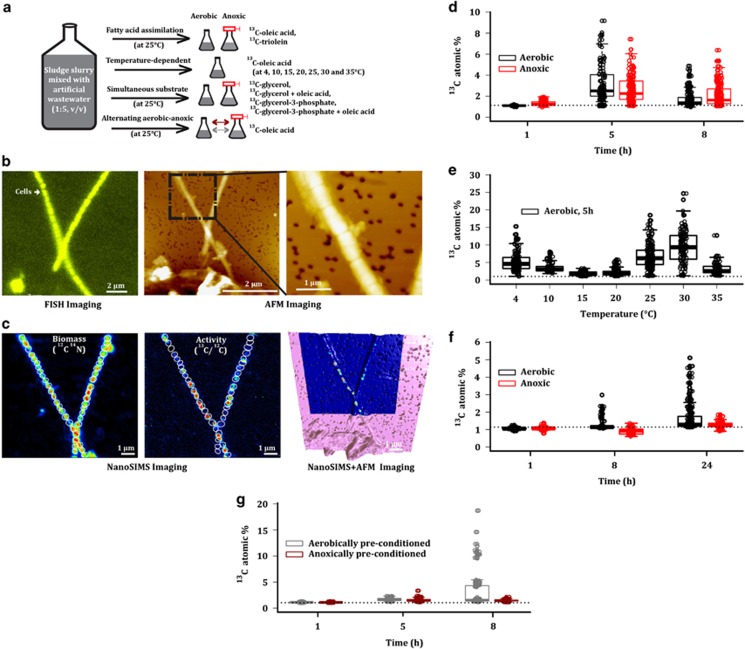
*In situ* phenotypic heterogeneity in substrate assimilation by ‘*Ca.* M. parvicella'. (**a**) Overview of the four independent isotopic incubation experiments. All experiments were conducted at 25 °C, except for the temperature-dependent experiment for which various temperature ranges were used. (**b**) Fluorescence *in situ* hybridization (FISH) with a ‘*Ca*. M. parvicella'-specific probe followed by atomic force microscopy (AFM) imaging to verify cellular integrity among ‘*Ca.* M. parvicella' cells. (**c**) The same region was analyzed using nanoSIMS to obtain ^13^C-isotopic enrichment information. AFM and nanoSIMS images were overlayed to highlight the distribution of newly assimilated substrates among ‘*Ca*. M. parvicella' cells. Regions of interest around individual ‘*Ca*. M. parvicella' cells were defined manually using the corresponding FISH images and their corresponding ^13^C atomic percentages were subsequently calculated. (**d**) ^13^C-oleic acid assimilation at different time points under either aerobic or anoxic conditions. (**e**) Temperature-dependent aerobic assimilation of ^13^C-oleic acid by single cells of ‘*Ca*. M. parvicella' after 5 h of incubation. (**f**) ^13^C-glycerol-3-phosphate assimilation under aerobic or anoxic conditions when administered as a single substrate. (**g**) Assimilation of ^13^C-oleic acid following alternating aerobic–anoxic conditions. (**d**–**g**) The dotted line indicates the ^13^C atomic percentage of ‘*Ca*. M. parvicella' single cells from time point 0 h.

**Figure 2:**
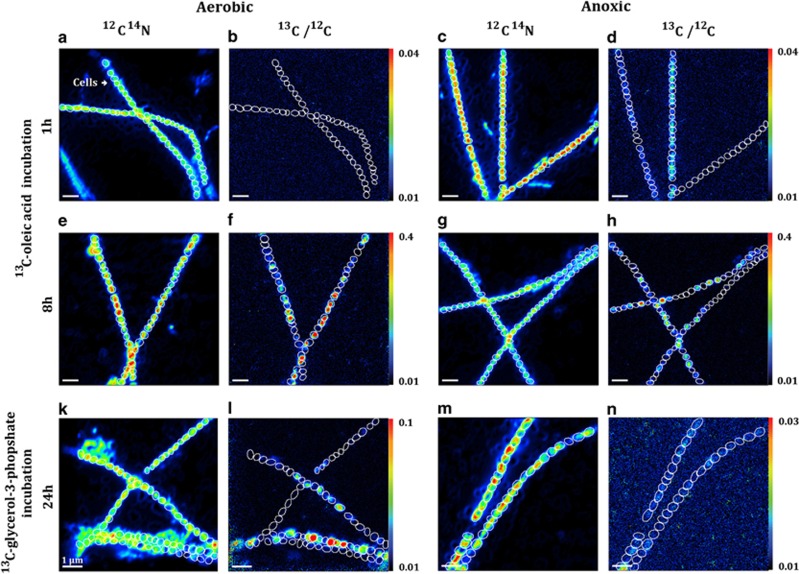
NanoSIMS visualization of phenotypic heterogeneity with regard to substrate assimilation among “*Ca.* M. parvicella” filaments under aerobic or anoxic conditions. The micrographs show ^13^C-oleic acid assimilation after 1 h (**a**–**d**) and 8 h during the fatty acid assimilation experiment (**e**–**h**) and ^13^C-glycerol-3-phosphate after 24 h when administered as a single substrate during the simultaneous substrate assimilation experiment (**k**–**n**).
