# Supplementary material for: In situ phenotypic heterogeneity among single cells of the filamentous bacterium Candidatus Microthrix parvicella
Source: ISME J. 2015 Oct 27;10(5):1274–9. doi: 10.1038/ismej.2015.181 (PMC5029219; doi:10.1038/ismej.2015.181)
Supplement: Supplementary Materials and Methods [file ismej2015181x1.pdf]

Supplementary information

In situ phenotypic heterogeneity among single cells of the filamentous bacterium *Candidatus* Microthrix parvicella

Abdul R. Sheik[1], Emilie E.L. Muller[1], Jean-Nicolas Audinot[2], Laura A. Lebrun[1], Patrick Grysan[2], Cedric Guignard[3], Paul Wilmes[1]

[1]Luxembourg Centre for Systems Biomedicine, University of Luxembourg, Esch-sur-Alzette, Luxembourg.

[2] Materials Research and Technology Department, Luxembourg Institute of Science and Technology, 41 rue du Brill, L-4422, Belvaux, Luxembourg.

[3] Department of Environmental Research and Innovation, Luxembourg Institute of Science and Technology, 41 rue du Brill, L-4422, Belvaux, Luxembourg.

Correspondence: A R Sheik, Luxembourg Centre for Systems Biomedicine, University of Luxembourg, 7, avenue des Hauts-Fourneaux, L-4362 Esch-sur-Alzette, Luxembourg. E-mail: abdul.sheik@uni.lu;

P Wilmes, Luxembourg Centre for Systems Biomedicine, University of Luxembourg, 7, avenue des Hauts-Fourneaux, L-4362 Esch-sur-Alzette, Luxembourg. E-mail: paul.wilmes@uni.lu

Supplementary Methods

Four independent isotopic incubation experiments were performed to investigate the substrate assimilation patterns by *Candidatus* Microthrix parvicella (*M. parvicella*). At first, we performed a fatty acid assimilation experiment using ^{13}C-triolein and ^{13}C-oleic acid under aerobic and anoxic conditions, respectively. Following these experiments, ^{13}C-oleic acid assimilation was investigated at different temperatures (4, 10, 15, 20, 25, 30 and 35°C). Then, we performed experiments in which ^{13}C-glycerol or ^{13}C-glycerol-3-phosphate were administered in combination with or without unlabeled oleic acid. Lastly, we performed incubation experiment with hourly alternating aerobic-anoxic conditions. All of the different experimental regimes were conducted in a similar fashion as described below. Except for the temperature-dependent experiment, all of the experiments were conducted at 25°C.

Experimental setup

For each experiment, fresh activated sludge was collected from a municipal biological wastewater treatment plant located in Schifflange, Esch-sur-Alzette, Luxembourg (Roume *et al.*, 2013; Muller *et al.*, 2014). After sampling, the sludge was immediately transferred to the laboratory within 15 minutes. The sludge was then mixed with artificial wastewater at a ratio of 1:5 (v/v) to obtain a final volume of 2 l. The mineral composition of artificial wastewater as described by Slijkhuis (Slijkhuis, 1983a), which has been previously used to study physiological characteristics of *M. parvicella* at varying oxygen concentrations and temperatures (Slijkhuis, 1983b, 1983a). The artificial wastewater contained $MgSO_4.7H_20$, 0.075 g l^{-1}; $CaCl_2$, 0.05 g l^{-1}; K_2HPO4, 8.2 g l^{-1}; KH_2PO_4, 0.35 g l^{-1}; $FeCl_3.6H_20$, 5 mg l^{-1}; $MnSO_4.H_20$, 6 mg l^{-1}; $ZnSO_4.7H_20$, 0.2 mg l^{-1}; $CuSO_4.5H_20$, 0.2 mg l^{-1}; H_3BO_3, 0.2 mg l^{-1}; $Na_2MoO_4.2H_20$, 0.1 mg l^{-1}; $CoCl_2.6H_20$, 0.1 mg l^{-1} and 10 ml l^{-1} of vitamin stock solution. We performed a 1:5 (v/v) dilution of the sludge biomass with artificial wastewater to reduce ambient lipid substrates and to facilitate

assimilation of added isotopic substrate by *M. parvicella* cells. Furthermore, considering the changes in the substrate composition of incoming wastewater, usage of artificial wastewater facilitates experimental reproducibility of independently conducted incubations. The 2 l mixed sludge was split into two 1 l aliquots which were pre-conditioned to aerobic or anoxic conditions for two hours. Anoxic incubations were performed inside an environment-controlled glove box (Jacomex, Dagneux, France), into which nitrogen gas was constantly flushed to achieve a gaseous dioxygen-free environment (<1000 ppm) which was continuously monitored using an oxygen sensor probe (OX-2, TCPS, Rotselaar, Belgium).

For each incubation experiment, 250 ml of each 1 l of either aerobically or anoxically incubated sludge was transferred into 300 ml Erlenmeyer flasks. Experimental sampling was carried out soon after the amendment of 500 µM of ^{13}C-substrates (Cambridge Isotope Laboratories INC., Andover, MA, USA) and nutrients at a final concentration of 80 µM NO_3^- and 16 µM PO_4^{2-}. All isotopically labelled substrates were suspended in 100 ppm Triton X-100 as described previously (Andreasen and Nielsen, 1997). ^{13}C-oleic acid, ^{13}C-glycerol and ^{13}C-glycerol-3-phosphate were uniformly labeled, whereas for ^{13}C-triolein only three carbon atoms were isotopically labeled. Experiments were performed in duplicates and samples were taken frequently at 0, 1, 2, 3, 5, 8, 12, 18, 24, and 30h post to the addition of the respective isotopic substrates.

Alternating aerobic-anoxic phases experiment

We performed an experiment involving alternating aerobic-anoxic phases to further investigate phenotypic heterogeneity in relation to changing environmental conditions typically encountered in a BWWTP. Two liters of freshly sampled sludge biomass diluted with 1:5 (v/v) artificial wastewater were prepared as described above. 30 ml of this mixture were transferred into eight 50 ml serum vials and were sealed using a sterile rubber stopper. Four of these serum vials were connected to a multi-fold valve system

(Discofix 5SM, Braun, Melsungen, Germany) and compressed air was bubbled through the mixture to establish aerobic conditions. Similarly, nitrogen gas was bubbled through the rest of the four serum vials to achieve anoxic conditions therein. To facilitate easy alternation between aerobic to anoxic conditions and vice-versa, two of the serum vials, preconditioned under either aerobic or anoxic conditions, were connected using a T-junction connector (BD Connecta, Helsingborg, Sweden). Meanwhile, the rest of two serum vials from both conditions were uninterruptedly purged either with air or nitrogen gas throughout the experimental regime. Experimental vials were preconditioned for 2h to their respective conditions, i.e. either aerobically or anoxically. The aerobic-anoxic and anoxic-aerobic alternations were started immediately after the addition of ^{13}C-oleic acid along with nutrients. Sampling was carried out on an hourly basis from 0 to 8h.

Fluorescence in situ *hybridization (FISH) and laser marking*

To identify *M. parvicella* cells, FISH was performed according to standard protocols (Amann, 1995; Daims *et al.*, 2005). Briefly, samples taken at each time point were fixed immediately in 4 % w/v paraformaldehyde, incubated overnight at 4°C, pelleted by centrifugation at 14000 *g*, washed three times in 1X PBS, resuspended in 1:1 (v/v) mixture of 1X phosphate buffer saline (PBS) and absolute ethanol, and stored at -20°C until further analyses. For FISH, 25 µl of pelleted biomass was resuspended in 10 ml of 1X PBS and filtered onto white polycarbonate membrane filters (GTTP, 0.2 µm pore size, Millipore), washed with 5-10 ml of ultrapure water (MQ, Millipore), air-dried and kept at room temperature until processing. Filter samples were excised using a 10 mm diameter round stencil and were then hybridized with the MPA60 probe specific for *M. parvicella* (Erhart *et al* 1997). Hybridized filter pieces were counterstained with a 1 µg ml^{-1} solution of 4,6-diamidino-2-phenylindole (DAPI).

Prior to atomic force microscopy (AFM) and nano-scale secondary-ion mass spectrometry (nanoSIMS) analyses, excised filter pieces containing *M. parvicella* biomass where laser etched using a laser micro-dissection microscope (Leica, Berlin, Germany) to provide orientation guides. For each laser marked region, images of DAPI-stained and MPA60 hybridized *M. parvicella* cells were first acquired using epifluorescence microscopy (Nikon wide-field microscope, Japan). These regions were then used as references for the subsequent AFM and NanoSIMS images.

AFM analyses

AFM analyses were performed with a PicoLE microscope (Molecular Imaging, Ann Arbor, Michigan, USA) and images were acquired in air in AC mode at scan rates between 0.5 and 1 Hz. We used a semi-contact silicon cantilever (RTESP; Bruker, Camarillo, CA, USA) with a spring constant of 40 $N.m^{-1}$. The surface topography of filaments and cells was acquired by height channel and images were processed with the manual tilt correction of the SPIP software (ImageMet).

Cell viability assays

Our assessment of cell viability using AFM imaging was based on the consideration that membrane integrity allows distinction between viable cells (consisting of assimilating and non-assimilating cells) and dead cells (Grégori *et al.*, 2001; Amann and Fuchs, 2008). Furthermore, Live-Dead staining (Boulos *et al.*, 1999) was conducted following the manufacturer's guidelines using freshly sampled biomass from aerobic, anoxic and alternating aerobic-anoxic conditions to assess the viability of *M. parvicella* cells as previously described (Roume *et al.*, 2013). Images of Live-Dead-stained *M. parvicella* cells were acquired using epifluorescence microscopy (Nikon wide-field microscope, Japan).

NanoSIMS analyses

Prior to nanoSIMS analyses, excised filter pieces were sputter-coated with gold (20 nm diameter). The laser-etched orientation guides on the excised filter pieces were identified with the charge-coupled device (CCD) camera on the nanoSIMS 50 instrument (Cameca, Gennevilliers, France). NanoSIMS analyses were performed on the samples derived from one replicate of each experimental regime. NanoSIMS analyses were performed on the previously marked regions which were oriented to correspond with the FISH images. The primary ion beam had a nominal size of between 50 and 100 nm and the image was the sum of acquisition of 30 images acquired with a dwelling time of 2 ms per pixel. The primary current of the Cs^+ beam was 180 pA during pre-sputtering and 1.2 – 1.5 pA during acquisition for most images and a raster size of 8 x 8 μm^2 or 10 x 10 μm^2. For each analysis, we recorded simultaneously secondary-ion images of naturally abundant ^{12}C (measured as $^{12}C^-$), ^{14}N (measured as $^{12}C^{14}N^-$) for distribution of biomass and similarly ^{13}C (measured as $^{13}C^-$) for the substrate assimilation quantification and growth rates estimation. NanoSIMS data-sets were analyzed using the Look@NanoSIMS software (Polerecky *et al.*, 2012). *M. parvicella* filaments and single cells along were identified using FISH images taken prior to nanoSIMS analyses. Regions of interest (ROI) around individual *M. parvicella* cells were defined manually using FISH image. The isotope ratio (r= $^{13}C/^{12}C$) was calculated for each ROI based on the total $^{13}C^-$ and $^{12}C^-$ counts for each pixel. Subsequently, the ^{13}C atomic percentage was calculated as $^{13}C*100/(^{13}C+^{12}C)$.

Bulk analyses and growth rate calculations

Bulk concentrations of ^{13}C-oleic acid and ^{12}C-oleic acid and long-chain fatty acids such as ^{12}C-mono-olein, ^{12}C-di-olein, ^{12}C-tri-olein in the supernatant fraction and ^{13}C-glyceryl trioleate in the biomass fraction were quantified using liquid chromatography coupled to tandem mass spectrometry (LC-MS/MS). Compounds of interest were extracted using a biomolecular extraction protocol as described previously by Roume *et al* (2013).

Target compound separation and detection were achieved by LC (1260SL Agilent, Germany) coupled to tandem mass spectrometry (4500 QTrap, AB/Sciex, The Netherlands) with electrospray ionisation in multiple reaction monitoring (MRM) in negative mode. For separation, a Kinetex C18 column (Phenomenex) was used (100x 2.1mm) with a mobile phase consisting of ethyl acetate and methanol with 10mM of ammonium acetate in a linear gradient.

The growth rates (d^{-1}) of *M. parvicella* cells were calculated based on ^{13}C atomic percentage and the labeling percentage of the amended isotopic substrate as described by Foster *et al* (2011).

Growth rates (d^{-1}) were calculated by the following:

$$\mathrm{V} = \left(1/t\right)\times\frac{\left(Rf - Ri\right)}{\left(Rs - Ri\right)}$$

where t is time in day, the R_f is estimated from the ^{13}C atomic percentage of *M. parvicella* cells at a specific time point by nanoSIMS, the R_i is the ^{13}C atomic percentage of *M. parvicella* cells from the time zero samples, and the R_s is the calculated labeling percentage of ^{13}C-substrate in the experimental flask.

References

Amann R, Fuchs BM. (2008). Single-cell identification in microbial communities by improved fluorescence in situ hybridization techniques. *Nat Rev Microbiol* **6**:339–48.

Amann RI. (1995). *In situ* identification of micro-organisms by whole cell hybridization with rRNA-targeted nucleic acid probes. In:*Molecular microbial ecology manual*, Springer, pp. 331–345.

Andreasen K, Nielsen PH. (1997). Application of microautoradiography to the study of substrate uptake by filamentous microorganisms in activated sludge. *Appl Environ Microbiol* **63**:3662–3668.

Boulos L, Prevost M, Barbeau B, Coallier J, Desjardins R. (1999). LIVE/DEAD® BacLight™: application of a new rapid staining method for direct enumeration of viable and total bacteria in drinking water. *J Microbiol Methods* **37**:77–86.

Daims H, Stoecker K, Wagner M. (2005). Fluorescence in situ hybridization for the detection of prokaryotes. *Mol Microb Ecol* **213**:239.

Erhart R, Bradford D, Seviour RJ, Amann R, Blackall LL. (1997). Development and use of fluorescent *in situ* hybridization probes for the detection and identification of *Microthrix parvicella* in activated sludge. *Syst Appl Microbiol* **20**:310–318.

Foster RA, Kuypers MMM, Vagner T, Paerl RW, Musat N, Zehr JP. (2011). Nitrogen fixation and transfer in open ocean diatom-cyanobacterial symbioses. *ISME J* **5**:1484–93.

Grégori G, Citterio S, Ghiani A, Labra M, Sgorbati S, Brown S, *et al.* (2001). Resolution of viable and membrane-compromised bacteria in freshwater and marine waters based on analytical flow cytometry and nucleic acid double staining. *Appl Environ Microbiol* **67**:4662–4670.

Muller EEL, Pinel N, Laczny CC, Hoopmann MR, Narayanasamy S, Lebrun LA, *et al.* (2014). Community-integrated omics links dominance of a microbial generalist to fine-tuned resource usage. *Nat Commun* **5**:5603.

Polerecky L, Adam B, Milucka J, Musat N, Vagner T, Kuypers MMM. (2012). Look@NanoSIMS – a tool for the analysis of nanoSIMS data in environmental microbiology. *Environ Microbiol* **14**:1009–23.

Roume H, Muller EEL, Cordes T, Renaut J, Hiller K, Wilmes P. (2013). A biomolecular isolation framework for eco-systems biology. *ISME J* **7**:110–121.

Slijkhuis H. (1983a). *Microthrix parvicella*, a filamentous bacterium isolated from activated sludge: cultivation in a chemically defined medium. *Appl Environ Microbiol* **46**:832–9.

Slijkhuis H. (1983b). The physiology of the filamentous bacterium *Microthrix parvicella*. PhD Thesis, Landbouwhogeschool, Wageningen, The Netherlands. http://library.wur.nl/WebQuery/wurpubs/77533.
