# Supplementary material for: In situ phenotypic heterogeneity among single cells of the filamentous bacterium Candidatus Microthrix parvicella
Source: ISME J. 2015 Oct 27;10(5):1274–9. doi: 10.1038/ismej.2015.181 (PMC5029219; doi:10.1038/ismej.2015.181)
Supplement: Supplementary Figures [file ismej2015181x2.pdf]

Supplementary Figure 1:

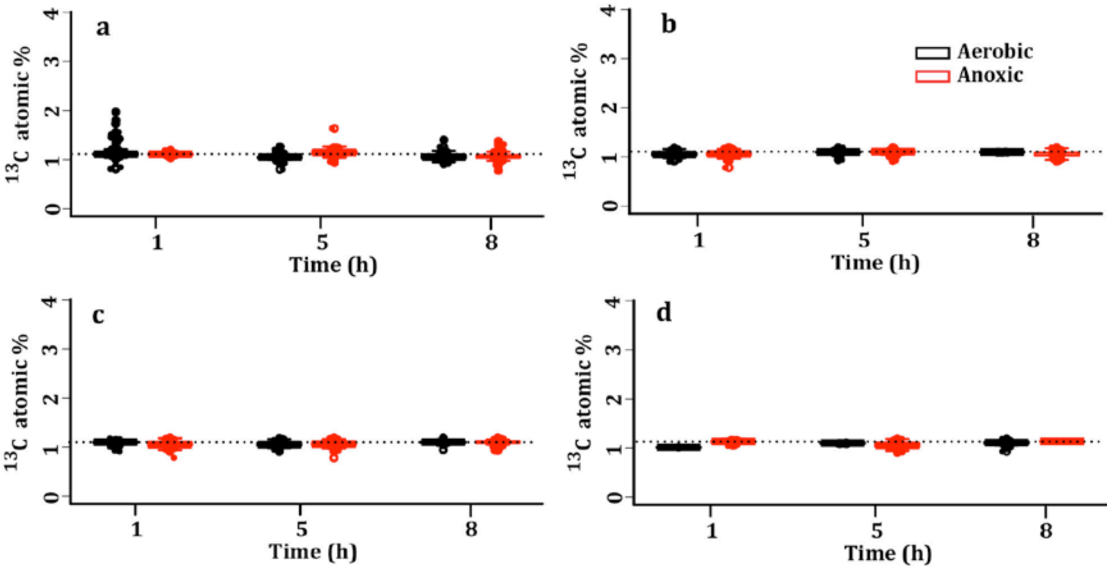

Supplementary Figure 1. Single-cell assimilation rates deduced from nanoSIMS analyses of *M. parvicella* cells following (a) ^{13}C-triolein, (b) ^{13}C-glycerol, (c) ^{13}C-glycerol and oleic acid, (d) ^{13}C-glycerol-3-phosphate and oleic acid incubations under aerobic and anoxic conditions.

Supplementary Figure 2:

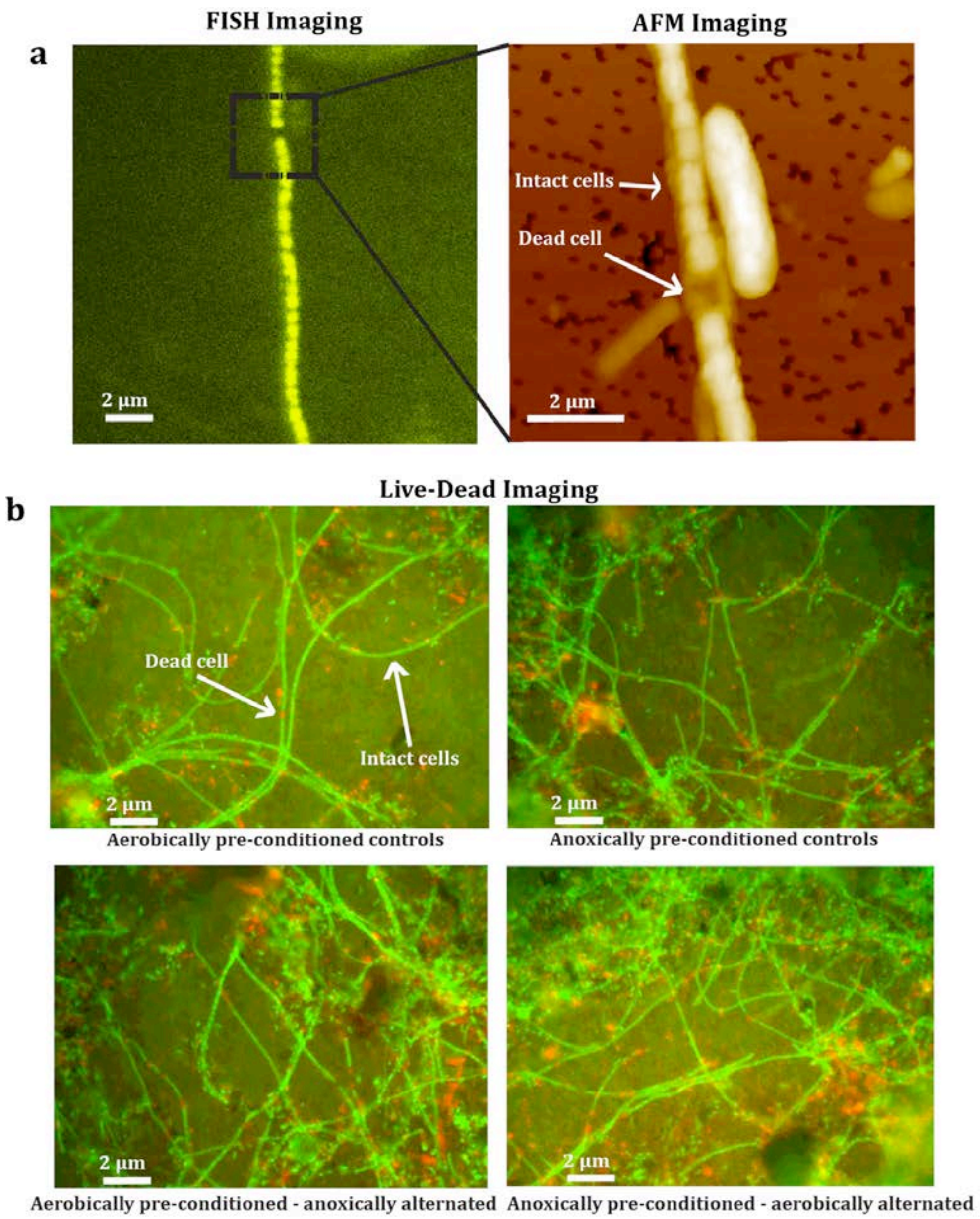

Supplementary Figure 2. Assessment of *M. parvicella* cell viability. (a) AFM imaging visualizing intact and dead *M. parvicella* cells. (b) Micrographs from Live-Dead staining highlighting the viability of experimental biomass in response to aerobic, anoxic and alternating aerobic-anoxic conditions following 8h of incubation. Green signals indicate viable cells whereas red signals indicate dead cells.

Supplementary Figure 3:

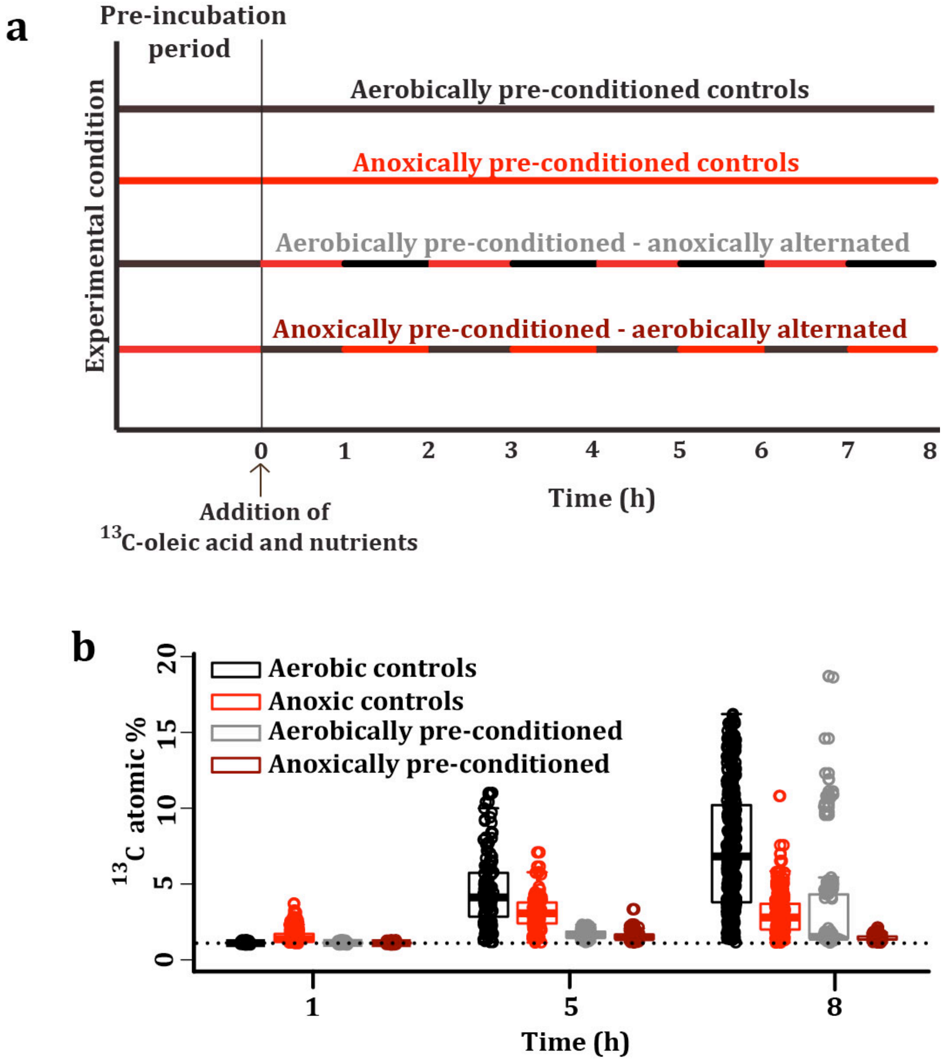

Supplementary Figure 3. (a) Schematic representation of the alternating aerobic-anoxic phases experiment. (b) NanoSIMS deduced single-cell assimilation rates of *M. parvicella*.
