# Supplementary material for: In situ phenotypic heterogeneity among single cells of the filamentous bacterium Candidatus Microthrix parvicella
Source: ISME J. 2015 Oct 27;10(5):1274–9. doi: 10.1038/ismej.2015.181 (PMC5029219; doi:10.1038/ismej.2015.181)
Supplement: Supplementary Table 1 [file ismej2015181x3.pdf]

Supplementary Table 1:

LC-MS/MS analysis results from the fatty acid assimilation experiment which involved quantification of bulk concentrations of lipid substrates from the supernatant and biomass fractions.
Error bars indicate standard deviation of the duplicates.
The concentrations of other long-chain fatty acids were below the detection limit of the instrument and therefore not presented.

	Supernatant fraction						Accumulation of newly assimilated ^{13}C-oleic acid within biomass fraction	
Time (h)	^{13}C-oleic acid (µM)		^{12}C-oleic acid (µM)		^{12}C-Triolein (µM)		^{13}C-Glyceryl trioleate (µM)	
	**Aerobic**	**Anoxic**	**Aerobic**	**Anoxic**	**Aerobic**	**Anoxic**	**Aerobic**	**Anoxic**
**0**	503.77 ± 58.15	515.07 ± 47.60	13.28 ± 1.19	13.48 ± 0.84	1.45 ± 0.34	1.57 ± 0.17	0.00	0.00
**1**	401.67 ± 50.80	358.28 ± 44.11	12.50 ± 0.89	10.19 ± 4.12	2.16 ± 1.35	2.25 ± 0.24	16.03 ± 3.87	15.67 ± 4.20
**5**	206.78 ± 46.89	175.52 ± 33.81	12.49 ± 2.30	10.72 ± 0.67	0.65 ± 0.04	0.65 ± 0.04	9.83 ± 0.64	15.11 ± 6.51
**8**	141.41 ± 31.17	72.02 ± 11.46	7.73 ± 1.45	12.26 ± 1.85	0.76 ± 0.21	0.81 ± 0.13	9.49 ± 2.98	5.91 ± 0.27
**20**	82.64 ± 19.87	10.18 ± 2.45	8.23 ± 0.62	10.51 ± 0.33	0.64 ± 0.02	0.66 ± 0.04	7.80 ± 0.23	0.22 ± 0.02
**30**	34.47 ± 8.29	n.d*	8.92 ± 0.78	13.13 ± 0.84	0.75 ± 0.18	0.69 ± 0.08	n.d	0.17 ± 0.01
**48**	20.45 ± 4.92	n.d	9.62 ± 0.94	12.66 ± 0.39	0.70 ± 0.26	0.46 ± 0.08	n.d	0.13 ± 0.04

n.d* Not detected
