# Supplementary material for: In situ phenotypic heterogeneity among single cells of the filamentous bacterium Candidatus Microthrix parvicella
Source: ISME J. 2015 Oct 27;10(5):1274–9. doi: 10.1038/ismej.2015.181 (PMC5029219; doi:10.1038/ismej.2015.181)
Supplement: Supplementary Table 2 [file ismej2015181x4.pdf]

Supplementary Table 2: Results of the quantification of phenotypic heterogeneity and different growth rates among *M. parvicella* cells.

Fatty acid assimilation experiment

^{13}C-oleic acid incubation

	1h		5h		8h	
	Aerobic	Anoxic	Aerobic	Anoxic	Aerobic	Anoxic
Number of cells analyzed	137	199	151	160	162	194
Number of non-assimilating cells	136	92	67	72	106	124
^{13}C-enriched cells (%)	0.7	53.8	55.4	55.0	34.6	36.1
Growth rate (d^{-1})	-	0.39 ± 0.01	0.62 ± 0.04	0.48 ± 0.02	0.18 ± 0.02	0.15 ± 0.02

^{13}C-triolein incubation

	1h		5h		8h	
	Aerobic	Anoxic	Aerobic	Anoxic	Aerobic	Anoxic
Number of cells analyzed	241	228	183	140	211	286
Number of non-assimilating cells	224	228	181	121	181	276
^{13}C-enriched cells (%)	7.1	0.0	1.1	13.6	14.2	3.5
Growth rate (d^{-1})	-	-	-	-	-	-

Temperature-dependent experiment

^{13}C-oleic acid incubation

Temperature (°C)	4	10	15	20	25	30	35
Number of cells analyzed	147	178	124	136	191	121	190
Number of non-assimilating cells	36	52	82	94	70	35	97
^{13}C-enriched cells (%)	75.2	71.0	33.9	30.9	63.2	70.9	48.9
Growth rate (d^{-1})	0.35 ± 0.02	0.21 ± 0.01	0.12 ± 0.01	0.19 ± 0.01	0.50 ± 0.02	0.78 ± 0.03	0.27 ± 0.02

Simultaneous substrate assimilation experiment

^{13}C-glycerol 3-phosphate

	8h		24h	
	Aerobic	Anoxic	Aerobic	Anoxic
Number of cells analyzed	198	190	200	134
Number of non-assimilating cells	188	177	158	91
^{13}C-enriched cells (%)	5.1	6.7	21.0	32.0
Growth rate (d^{-1})	-	-	0.73 ± 0.15	0.20 ± 0.02

Alternating aerobic-anoxic experiment

^{13}C-oleic acid incubation

	1h				5h				8h			
	Aerobic	Anoxic	Aerobic adapted	Anoxic adapted	Aerobic	Anoxic	Aerobic adapted	Anoxic adapted	Aerobic	Anoxic	Aerobic adapted	Anoxic adapted
Number of cells analyzed	273	156	288	274	138	83	202	131	360	255	165	340
Number of non-assimilating cells	270	69	286	272	48	43	177	111	98	123	119	330
^{13}C-enriched cells (%)	1.0	56.0	0.8	0.6	65.4	48.2	12.4	15.3	72.8	51.8	27.9	2.9
Growth rate (d^{-1})	-	0.48 ±0.04	-	-	0.68 ±0.04	0.53 ± 0.02	-	-	0.41 ± 0.01	0.13 ± 0.01	0.12 ± 0.02	-
